# Evaluation of an Emergency Department Influenza Vaccination Program: Uptake Factors and Opportunities

**DOI:** 10.5811/westjem.2022.5.55227

**Published:** 2022-08-19

**Authors:** Canada Parrish, Crystal A. Phares, Tim Fredrickson, John B. Lynch, Lauren K. Whiteside, Herbert C. Duber

**Affiliations:** * University of Washington, Department of Emergency Medicine, Seattle, Washington; † Harborview Medical Center, Department of Emergency Medicine, Seattle, Washington; ‡ University of Washington, Division of Allergy and Infectious Disease, Seattle, Washington

## Abstract

**Introduction:**

Influenza vaccines are commonly provided through community health events and primary care appointments. However, acute unscheduled healthcare visits such as emergency department (ED) visits are increasingly viewed as important vaccination opportunities. Emergency departments may be well-positioned to complement broader public health efforts with integrated vaccination programs.

**Methods:**

We studied an ED-based influenza vaccination initiative in an urban hospital and examined patient-level factors associated with screening and vaccination uptake. Our analyses included patient visits to the ED from October 1, 2019–April 1, 2020.

**Results:**

The influenza screening and vaccination program proved feasible. Of the 20,878 ED visits that occurred within the study period, 3,565 (17.1%) included a screening for influenza vaccine eligibility; a small proportion (11.5%) of the patients seen had multiple screenings. Among the patients screened eligible for the vaccine, 916 ultimately received an influenza vaccination while in the ED (43.7% of eligible patients). There was significant variability in the characteristics of patients who were and were not screened and vaccinated. Age, gender, race, preferred language, and receipt of a flu vaccine in prior years were associated with screening and/or receiving a vaccine in the ED.

**Conclusion:**

Vaccination programs in the ED can boost community vaccination rates and play a role in both preventing and treating current and future vaccine-preventable public health crises, although efforts must be made to deliver services equitably.

## INTRODUCTION

Prior to the COVID-19 pandemic (respiratory disease caused by a coronavirus, SARS-CoV-2, discovered in 2019), seasonal influenza was the most common vaccine preventable illness in the United States.The US Centers for Disease Control and Prevention estimates from the 2019–2020 influenza season suggest 39–56 million infections, 410,000–740,000 hospitalizations, and 24,000–62,000 deaths due to influenza.^1^ Despite this toll, less than half of the adult population received an influenza vaccine.^2^ Further, there are disproportionately low rates of vaccination among communities of color with less access to traditional healthcare services.^2^

Influenza vaccines are commonly provided through community health events and primary care appointments. However, acute unscheduled healthcare visits such as emergency department (ED) visits are increasingly viewed as important vaccination opportunities.^3^ With more than 145 million ED visits per year in the US, and a patient population that often includes vulnerable and underserved individuals/communities, the ED is uniquely positioned to support a comprehensive national vaccine strategy. Developing an effective ED-based vaccination program is complicated. Emergency physicians and other clinicians have limited time and may frequently opt out of screening for non-emergency concerns. Incorporating public health interventions into routine ED flow using the full spectrum of ED staff has the potential to reduce the burden associated with these non-emergent tasks and increase vaccination rates. In this retrospective, observational study we examined the uptake of a nurse-initiated influenza vaccination program in a single-center, urban ED during the 2019–2020 influenza season and explored patient-level correlates of screening participation and vaccination.

## METHODS

Harborview Medical Center (HMC) is a large, publicly owned, urban, Level I trauma center located on the West Coast with a mission focused on underserved, immigrant, and other vulnerable populations. During the 2019–2020 influenza season, HMC implemented a nurse-initiated ED vaccination program using a “task list” embedded in the electronic health record (EHR). The EHR screening tool used in this study was part of a non-interruptive, non-mandatory nursing task. The task list was used since it was easy to program and allowed nursing staff the flexibility requested by nursing leadership. It should be noted that this work was implemented just prior to an EHR change, and limited information technology capacity was available for a more integrated format. Nurse managers educated staff about the task list and how to use it. It was not included in the standard triage process, although individual nurses could choose to do this based on ED volume and wait times.

When the influenza task was selected, the nurse was prompted to ask a set of vaccine eligibility and exclusion criteria questions. Patients were considered eligible if they had not previously received the 2019–2020 flu season vaccine and there were no medical contraindications for vaccination (eg, enrolled in immunotherapy.). If eligible, the patient was asked whether they would like the influenza vaccine, which was then administered by the screening nurse if the patient consented. Information on eligibility, refusal, and vaccine administration were documented in the EHR.

Our analyses included all patient visits with Emergency Severity Index (ESI) ≥3 to the HMC ED from October 1, 2019–April 1, 2020. The ESI is a triage algorithm that classifies patients into five case groups ranging from 1 (most urgent) to 5 (least urgent) based on clinical acuity and resource needs.^4^ Those with ESI <3 were excluded, as screening for influenza vaccine eligibility in these patients may not be likely, feasible, or appropriate. Deidentified patient data was obtained by a data analyst from the electronic data warehouse. Variables analyzed for each visit in our descriptive analyses and regression modeling included the following: age as a binary variable categorized as <35 years of age (child though young adult, reference category) vs ≥35 years of age (middle-age to older adult); gender; insurance status; race/ethnicity; preferred language; designated primary care physician, and receipt of prior influenza vaccine.

We conducted two sets of analyses. In the first set, we used ED visits as the unit of analysis, comparing characteristics of those who were screened vs not screened during those visits. We then examined unique patients who were screened and eligible for vaccination, comparing characteristics among those who were ultimately vaccinated in the ED vs those who were not vaccinated among those screened eligible. For each set of analyses, we compared the distributions of patient characteristics using chi-square tests and constructed multivariable logistic regression models to examine patient factors associated with screening and vaccination uptake. Robust clustered standard errors by patient identifier were used to account for correlation across individual patients over time in the screening uptake models, as some patients had multiple ED visits within the study period.

## RESULTS

We included 20,878 ED visits from 13,765 unique patients in the analysis.

### Screening

During the study period 3,565 influenza vaccination-eligibility screenings (17.1% of all ED visits) occurred. Most patients who entered the ED had only one influenza vaccination screen; 11.5% of patients were screened more than once over the course of the study period. There were observed differences in key patient demographics between those screened and those not screened during the study period. Those who received screenings were slightly older and more likely to have English as a preferred language than those who did not get screened (Table 1). In our logistic regression models examining associations between patient characteristics and the performance of influenza vaccination-eligibility screening, we found statistically significant associations for age, Black race, and Asian race (Table 2). Age was associated with increased odds of being screened; patients who were Black or Asian had reduced odds of being screened.

### Vaccination

Among all 3,099 unique patients screened, 2,098 (67.7%) were deemed eligible for vaccination. Less than 1% of patients had documented contraindications. Of those 2,098 eligible, 916 ultimately received an influenza vaccination (43.7% of eligible patients). The remaining 1,182 patients declined vaccination as documented in the EHR after screening eligibility.

All patient characteristics included in our analysis, with the exception of race and insurance provider, were statistically significantly different between eligible screened patients who did and did not receive influenza vaccines in the ED (Table 1). The patient group that received an influenza vaccine had a higher mean age, lower proportion of females, higher proportion of Spanish speakers, and higher proportion of documented receipt of a prior influenza vaccine. In the multivariable regression models, the receipt of an influenza vaccine in the ED was positively associated with Spanish as preferred language, and documented evidence of prior influenza vaccination. There were statistically significant negative associations for patient gender and race. Vaccine-eligible patients who were female or Black had lower odds of receiving an influenza vaccine compared to male or White patients (Table 2).

## DISCUSSION

In this retrospective study, we found that an ED-based, nurse-initiated influenza vaccination program can be integrated into a busy clinical workflow. This program represents an opportunity to increase vaccination rates and support population health initiatives, especially for those with limited access to non-emergency care services.^5^ Important questions around workflow, financial viability, and limitations related to the scope and scale of potential non-emergent services performed in the ED setting still remain.

We observed substantial variability in patient characteristics between those who were screened for influenza vaccination eligibility, and subsequently those who eventually were immunized. Individuals who are Black or Asian were less likely to be screened for vaccine eligibility. While it is more difficult to comment on the disparities observed for vaccination uptake among eligible patients (this no longer represents a subgroup of all ED visits), we again note that vaccine-eligible Black patients were less likely to be vaccinated than White patients. Racial bias is well-documented in the American healthcare system generally^6^ and in the ED more specifically.^7^,^8^ This study again raises important questions about equity in healthcare delivery. Initiatives promoting vaccination in the ED must examine mechanisms that work toward consistent and equitable screening and vaccination to ensure that disparities in health services utilization are mitigated, not exacerbated. Additionally, future work should educate healthcare workers on how to talk to patients about vaccines including use of presumptive language^9^ and use of motivational interviewing as ways to address vaccine hesitancy.^10^

## LIMITATIONS

This study should be interpreted within the context of several important limitations. Perhaps most importantly, the EHR screening tool was not a part of standard, mandatory triage protocol, resulting in missed opportunities to screen and vaccinate a large number of ED patients. Integration into standard protocol could boost screening rates. Due to the nature of the data available for this analysis, we were unable to explore specific reasons as to why a patient was not screened or why a patient ultimately declined vaccination or had no documented vaccine. Further, our data does not include reason(s) for vaccine refusal nor information as to when or how healthcare workers provided motivation and counseling.

## CONCLUSION

Currently, the world is in the midst of the COVID-19 pandemic. Effective vaccines that protect against the severe consequences of COVID-19 exist, but critical thresholds of vaccination rates are needed to achieve sufficient levels of morbidity and mortality reduction in the population.^11^ The ED is uniquely poised to fill an important vaccination gap in reaching patients who are often vulnerable and lack access to primary care services.^12^ This work demonstrates that ED-based vaccination programs are feasibly implemented and can boost vaccination rates, though efforts must be made to ensure equitable delivery.

## Figures and Tables

**Table 1:** Descriptive characteristics of patients and emergency department visits, by screening status and vaccination status.

Patient characteristics	Screening status			Vaccination status (among those screened eligible)		
	
	Screened (n = 3,565)	Not screened (n = 17,313)	P-value	Vaccinated (n = 916)	Not vaccinated (n = 1,182)	P-value
Age in years, mean (SD)	46.2 (15.9)	45.4 (16.8)	**0.01**	44.2 (15.1)	42.8 (15.3)	0.04
Female, n (%)	1,269 (35.6)	6,006 (34.7)	0.30	279 (30.5)	431 (36.5)	**<0.01**
Race, n (%)			**0.01**			0.07
White	2,239 (62.8)	10,189 (58.9)		597 (65.2)	727 (61.5)	
Black	929 (26.1)	5,000 (28.8)		223 (24.3)	339 (28.7)	
Asian	195 (5.5)	1,107 (6.3)		40 (4.4)	53 (4.5)	
American Indian or Alaska Native	140 (3.9)	684 (4.0)		34 (3.7)	49 (4.2)	
Native Hawaiian or Pacific Islander	34 (1.0)	198 (1.1)		11 (1.2)	9 (0.8)	
Unknown or declined to answer	28 (0.8)	135 (0.8)		11 (1.2)	5 (0.4)	
Hispanic, n (%)	474 (13.3)	2,338 (13.5)	0.91	163 (17.8)	128 (10.8)	**<0.01**
Insurance, n (%)			0.75			0.09
Medicaid	1,642 (46.1)	8,104 (46.8)		446 (48.7)	591 (50.0)	
Medicare	812 (22.8)	3,894 (22.5)		160 (17.5)	210 (17.8)	
Commercial	456 (12.8)	2,089 (12.1)		100 (10.9)	160 (13.5)	
Self	469 (13.2)	2,325 (13.4)		155 (16.9)	157 (13.3)	
Other	186 (5.2)	901 (5.2)		55 (6.0)	64 (5.4)	
Language, n (%)			**0.02**			**<0.01**
English	3,136 (88.0)	14,980 (86.5)		770 (84.1)	1,075 (91.0)	
Spanish	225 (6.3)	1,125 (6.5)		88 (9.6)	50 (4.2)	
Other	204 (5.7)	1,208 (7.0)		58 (6.3)	57 (4.8)	
Designated PCP, n (%)	1,837 (51.5)	8,791 (50.8)	0.41	441 (48.1)	499 (42.2)	**0.01**
Prior influenza vaccine, n (%)	1,039 (29.1)	4,875 (28.2)	0.23	275 (30.0)	187 (15.8)	**<0.01**

*PCP*, primary care physician.

**Table 2:** Associations between patient characteristics and screening and vaccination status from adjusted analyses.

Patient characteristics	Screening status N = 20,878			Vaccination status (among those screened eligible) n = 2,098		
	
	Odds ratio	95% CI	P-value	Odds ratio	95% CI	P-value
Age, ≥ 35 years (ref < 35 years)	1.10	1.01, 1.20	0.04	1.06	0.87, 1.30	0.54
Female (ref male)	1.05	0.97, 1.13	0.26	0.70	0.58, 0.85	<0.01
Race (ref White)						
Black	0.85	0.77, 0.93	<0.01	0.76	0.61, 0.94	0.01
Asian	0.82	0.69, 0.97	0.02	0.86	0.54, 1.36	0.53
American Indian or Alaska Native	0.93	0.76, 1.14	0.48	0.84	0.53, 1.32	0.45
Native Hawaiian or Pacific Islander	0.79	0.54, 1.16	0.23	1.41	0.56, 3.55	0.46
Unknown or declined to answer	0.98	0.65, 1.47	0.91	2.19	0.72, 6.62	0.17
Hispanic (ref Non-Hispanic)	0.94	0.81, 1.09	0.43	1.30	0.93, 1.81	0.13
Insurance (ref Medicaid)						
Medicare	0.98	0.89, 1.09	0.76	0.86	0.66, 1.11	0.23
Commercial	1.07	0.95, 1.21	0.24	0.82	0.61, 1.10	0.18
Self	1.04	0.92, 1.18	0.53	1.07	0.80, 1.44	0.62
Other	1.02	0.86, 1.21	0.80	1.11	0.75, 1.65	0.58
Language (ref English)						
Spanish	0.91	0.79, 1.05	0.22	1.72	1.06, 2.79	0.02
Other	0.86	0.73, 1.01	0.08	1.54	1.02, 2.32	0.04
Designated PCP, n (%)	1.00	0.92, 1.09	0.99	1.10	0.91, 1.34	0.32
Prior influenza vaccine, n (%)	1.05	0.96, 1.16	0.26	2.33	1.85, 2.93	<0.01

*CI*, confidence interval; *ref*, reference; *PCP*, primary care physician
